# State Dependent Valuation: The Effect of Deprivation on Risk Preferences

**DOI:** 10.1371/journal.pone.0053978

**Published:** 2013-01-24

**Authors:** Dino J. Levy, Amalie C. Thavikulwat, Paul W. Glimcher

**Affiliations:** 1 Recanati Faculty of Management, Tel-Aviv University, Tel-Aviv, Israel; 2 Sagol School of Neuroscience, Tel-Aviv University, Tel-Aviv, Israel; 3 Center for Neural Science, New York University, New York, New York, United States of America; CNR, Italy

## Abstract

The internal state of an organism affects its choices. Previous studies in various non-human animals have demonstrated a complex, and in some cases non-monotonic, interaction between internal state and risk preferences. Our aim was to examine the systematic effects of deprivation on human decision-making across various reward types. Using both a non-parametric approach and a classical economic analysis, we asked whether the risk attitudes of human subjects towards money, food and water rewards would change as a function of their internal metabolic state. Our findings replicate some previous work suggesting that, *on average*, humans become more risk tolerant in their monetary decisions, as they get hungry. However, our specific approach allowed us to make two novel observations about the complex interaction between internal state and risk preferences. First, we found that the change in risk attitude induced by food deprivation is a general phenomenon, affecting attitudes towards both monetary and consumable rewards. But much more importantly, our data indicate that rather than each subject becoming more risk tolerant as previously hypothesized based on averaging across subjects, we found that as a population of human subjects becomes food deprived the heterogeneity of their risk attitudes collapses towards a fixed point. Thus subjects who show high-risk aversion while satiated shift towards moderate risk aversion when deprived but subjects who are risk tolerant become more risk averse. These findings demonstrate a more complicated interaction between internal state and risk preferences and raise some interesting implications for both day-to-day decisions and financial market structures.

## Introduction

Any animal that faces a changing environment needs to have the ability to acquire resources under variable environmental conditions and to structure their behavior in a way that makes efficient use of that variability. Particularly when resources become scarce, being sensitive to this variability may be of particular importance. Economists and foraging theorists often refer to the strategies that guide humans and animals in their exploitation of variable environments as risk attitudes, specific strategies for valuing rewards that make animals more or less conservative in their response to environmental variability. Critical to any efficient strategy with regard to risk is an organism's internal state. Many have hypothesized that a subject's response to a variable environment, if it is to be efficient, must include a modulation of risk attitudes by internal factors like food and water wealth or deprivation.

Traditional risk-sensitive foraging theory, to take one example, suggests that an organism's foraging decisions with regard to consumable rewards should depend upon satiation level, although it should be noted that the precise function that relates optimal risk-tolerance to satiation level remains a subject of significant normative debate [1]–[7]. One line of argument from this literature suggests that as an animal becomes so food or water deprived that death is immanent, it should become risk seeking, willing to gamble everything on a chance for food or water (e.g. [8]). Another line of reasoning, however, suggests just the opposite [4], [9]. Existing data is equally contested, with some data showing evidence of risk seeking under conditions or deprivation and other data showing just the opposite (for reviews see [4], [6], [10]). Of course this suggests that both normative theoretical and empirical behavioral studies point toward complex non-linear relationships between risk attitude and deprivation state [2].

Very recent data have extended these kinds of observations to human decision-makers operating in the monetary domain. Symmonds and colleagues [11] demonstrated that human risk attitudes towards money do in fact vary as a function of hunger-state. They showed that as the satiating effect of a meal increases, a population of humans becomes more risk averse in decisions about monetary rewards. However, Symmonds and colleagues did not examine the relationship between food deprivation and risk attitudes towards food or water but only towards money, nor did they estimate a risk attitude for each of their individual subjects.

The current study extends this previous work in five ways. First, we extended the findings of Symmonds and colleagues by demonstrating the extent to which mild food and water deprivation alters risk attitudes towards monetary rewards in humans using standard economic models of risk attitudes. Second, we determined how mild food and water deprivation alters risk attitudes towards food and water, a class of decisions widely studied in non-human animals but not in the Symmonds and colleagues study. This is of some relevance because we recently showed that the risk attitudes of individual human subjects to different reward types are highly correlated, although the risk attitudes across subjects in that population were highly heterogeneous [12]. Third, we assessed how the relative values of food, water and money (exchange rates) change as subjects become deprived. Fourth, and most important, we made within-subjects measurements adequate for describing how risk attitudes towards all three rewards change as a function of deprivation in individual subjects. Fifth, we used these individual-level assessments to examine the variability of risk attitudes within our subject population. We found that the variability of risk attitudes was a function of deprivation state.

**Figure 1:**
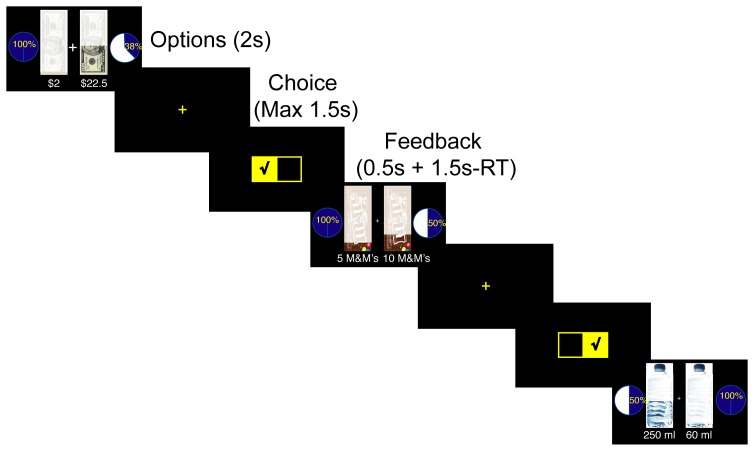
Trials timeline. On each trial two options were presented for two seconds. This presentation was followed by the appearance of a yellow cross, which signaled a maximum of 1.5 s for indicating the preferred option by pressing one of two buttons on a computer mouse. Thereafter, a feedback screen indicating the subject's choice was presented for 0.5 s plus the difference between 1.5 s and the reaction time (RT) to make sure that the total time of choice plus feedback was 2 s. This was followed immediately with the next trial.

As in many previous studies, we examined risk attitudes by asking human subjects to repeatedly choose between a small certain reward and a larger risky reward in a binary choice task. We presented three reward types: food, water and money, and systematically varied both the risk and magnitude associated with the larger reward (Figure 1). First, we conducted a non-parametric analysis of subject choices as a function of state without committing to any specific model of risk attitude. For this purpose we simply computed the proportion of times that each subject picked the uncertain of the two offered options. In a second approach, we used expected utility theory (EUT) and modeled the many choices of each subject as the product of a single parameter power law utility function that related subjective desirability to objective reward magnitude. This convenient single parameter model (α) represents each subject's risk attitude in a highly compact form. An α<1 represents risk aversion, α = 1 represents risk neutrality and α>1 represents risk seeking behavior. The third measure we generated determined the relative values of money/food and money/water for each subject in order to establish more completely how preferences change with deprivation. We employed this final measure to determine whether deprivation state affects risk preferences for each reward type independently or whether it also affects the interaction of relative values (the exchange rates) between reward types.

## Materials and Methods

### Subjects

A total of 97 subjects (65 women) were enrolled in this study and completed at least one behavioral session. All participants gave written informed consent. All procedures were in compliance with the safety guidelines for behavioral research and were approved by the University Committee on Activities Involving Human Subjects of New York University. Out of the 97 subjects, 56 subjects attended two sessions (satiated and deprived). Of the subjects who attended both sessions, parametric risk parameters (described below) could not be accurately estimated for one subject. This subject was discarded from all further analysis. All data reported here was gathered in the remaining 55 subjects (35 women).

### General procedure

Two sessions composed the study, satiated and deprived. In the deprived session participants were asked to refrain from eating and drinking for four hours prior to coming to the laboratory for testing. In the satiated session, participants were asked to eat a full lunch (including something to drink) immediately before testing. All sessions started between 10:00am to 1:00pm local time. Ninety percent of subjects started the experiment at 10:00am in both sessions and the other 10% started the experiment between 10:00am to 1:00pm. The order of the sessions was counterbalanced across subjects.

Money and two primary rewards (food and water) were offered to the subjects during experimental trials. Before the first experimental session began subjects were offered a choice between two food rewards: Small chocolate candies (*M&Ms*; Mars nutrition) or small salted crackers (*mini-Ritz*; Kraft foods). The food reward that they selected then served as the target of all future food choices for that subject. Of the 55 subjects 29 selected chocolate candies. Water offers were for a fixed number of milliliters of spring water. Monetary offers were in units of US dollars.

### Behavioral sessions

Before each session, subjects were asked to report their current hunger and thirst levels (“How hungry/thirsty are you right now”) using a visual analog scale (VAS). Subjects were then asked to perform 450 *same-type trials* (150 choices over each of the three rewards types; money, food and water) to assess risk aversion within a reward type and 300 *mixed-type trials* (150 choices over money-food lotteries and 150 choices over money-water lotteries) to assess the relative values of different kinds of rewards, in a total of 12 blocks. All trials were randomly interleaved. Subjects received $40 for completing each of the behavioral sessions, which lasted approximately 1 hour and during which the subjects made a total of 750 choices. Subjects were informed, in advance, that after testing they would be asked to remain in the laboratory for 2 hours during which the only food and water to which they would have access was the food and water realized from one trial of each type selected randomly at the end of the experiment.

On each trial, two options were presented on a computer screen for two seconds (Figure 1). This was followed by a yellow cross in the middle of the screen, signaling that the subject should indicate which option they preferred, by pressing one of two buttons on a computer mouse, within 1.5 s. A feedback screen indicating the subject's choice was presented for 0.5 s plus any remaining time in the 1.5 s response period. The next trial then followed immediately. Failing to make a choice within the given time resulted in an error signal during the feedback interval. Missed trials were not repeated. Of the 750 trials in a session subjects missed on average 10 trials (range: 0–50).

### Same-type trials

In *same-type trials* subjects were asked to choose between a certain small reward (the *reference* option) and a stated probability of either winning a larger amount of the same reward (money, food or water) or getting nothing (the *lottery* option). The value of the *reference* option during same-type trials was fixed throughout the experiment ($2, 5 chocolate candies or 2 salty crackers, and 60 ml of water). There were five different values for the *lottery* option for each reward type (2, 4.5, 10, 22.5, 50 dollars; 5, 10, 20, 40, 80 candies or 2, 5, 10, 20, 40 crackers and 60, 125, 250, 500, 1000 ml of water). Five different winning probabilities (13%, 22%, 38%, 50% and 75%) were fully crossed with these 5 reward magnitudes, yielding 25 unique lottery options for each of the 3 reward types. These 75 unique lottery options constituted 1 block of a session. Each choice pair was presented 6 times in 6 separate blocks in each session in randomized order for a total of 450 same-type trials per session. Same-type trials were designed to measure the risk preferences of each subject with regard to each of the three reward types independently.

### Mixed-type trials

In mixed-type trials subjects were asked to choose between a sure win of a small amount of money ($0.50) and a stated probability of either winning a fixed amount of food or water or getting nothing. Five amounts (10, 20, 30, 50, 80 candies or 5, 10, 15, 25, 40 crackers and 125, 250, 400, 600, 1000 ml of water) in the same range as in the same-type trials with the same 5 winning probabilities as in the same-type trials were used resulting in 25 unique lotteries for food and water. These 50 unique lottery options for food and water constructed 1 block within each session. Each unique choice was presented 6 times in 6 separate blocks in each session in randomized order, for a total of 300 mixed-type trials per session. Mixed type trials were designed to measure the relative values of the three reward types; to establish the subjective exchange rates for the three different kinds of rewards.

### Description of stimuli

The reward magnitude of each option was written numerically in the display and was also represented as a fraction revealed from a $50 bill in the **same**-type trials (or $0.50 in the **mixed**-type trials, a fraction revealed from a $1 bill), a pack of M&M's (40 pcs of candy), a pack of crackers (20 pcs of crackers) or a 500 ml bottle of water. The winning probability was explicitly stated numerically and represented as a fraction of a full circle.

### Realization of choices

At the conclusion of each session one, and only one, completed trial of each type (a total of 4 trials) was randomly selected and played for real money and/or real primary rewards.

If on a selected trial the subject had chosen the reference option, they received that amount of food, money or water. If on a selected trial the subject had chosen the lottery option, then a random number generator determined whether or not the subject had won (according to the winning probability of the selected trial) the specified amount of food, money or water. Subjects did not receive rewards as they performed the tasks nor were the lottery outcomes revealed to the subjects as tasks were being performed. At the conclusion of the realization process, subjects were given their consumable rewards and asked to stay in the lab for an additional two hours. During this period the only food and drink they were allowed to consume was what they had realized from the experiment. We imposed this 2 h delay for two reasons; first, it insured that the choices made by the subjects over consumable rewards had an impact on their physiological state over an extended period. Second, it insured that subjects could not effectively maximize their food and water intake on mixed trials by always selecting the monetary reward and then leaving the lab to purchase candy or crackers at market prices. Our observation that subjects typically valued the food and water rewards at 2–3 times their market value (as described in the results section) suggests that this manipulation was successful. All subjects studied remained in the lab for this additional two-hour period.

### Estimating Risk Preferences

We wanted to examine the effect of internal state on subjects' risk preferences. The first method we used was a non-parametric approach. In this approach we did not commit to any specific model of how risk attitudes should be represented but rather examined subjects' choices and computed the proportion of trials on which they chose the *lottery* option as a fraction of their total number of choices for each reward type in each session. This measure gives an estimate of subjects' propensity to choose the *lottery* option in each state and would be ordinally correlated with essentially all models that might be used to describe risk preference. The main question that we asked with this non-parametric analysis was whether internal state exerted a systematic effect on the propensity to choose the *lottery* option, i.e., their willingness to accept a risky outcome.

The second method that we used was (random) expected utility theory. Our goal was order to derive a utility function for each subject, for each reward type, in each session, using the data from the same-type trials. When subjects are consistent in their choices (as was the case in our data) the curvature of these utility functions serves as one common measure of their risk-preferences [9]. Of course many other models of risk preference are possible. We selected this model because i) it is widely used to describe risk attitudes, ii) it described the behavior of our subjects on these simple choices with very high fidelity, and iii) it yielded a single parameter that summarized risk-acceptance.

In both methods we used two procedures to analyze our data. The first was to pool the data of all subjects in each session and to determine the average risk preferences for our population. This is, of course, the *representative agent* approach common in economics and it is essentially the procedure used by Symmonds and colleagues in their examination of these same issues. Note, that for the representative agent analysis we have clustered the error term of the regression, using each of our subjects as a cluster, in order to account for within-subject trial dependency. The second analysis we used was to separately analyze the data for each subject in each reward type in each of the sessions using both the parametric and non-parametric approaches.

### Non-Parametric Method

We used a logistic regression with subjects' overall choices across all same reward type trials for money, food and water as the dependent variable and examined the effect internal state has on choice. Note that we ran this regression after pooling all the data of all subjects (clustering error by subject) and all reward types combined. We also ran the same regression but separately for each reward type (again, data was pooled across all subjects). This allowed us to examine the average effect of internal state on subjects' propensity to choose the risky option. Formally, we ran a logistic regression having the form:
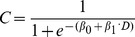
Where C represents subjects' choices (taking the value of 0 or 1 for *reference* or *lottery* option, respectively), *D* is the variable representing session type (deprived or satiated) and *β* is the slope of the logistic function. We tested if the coefficient of the state parameter *D* (*β*_1_) in the regression was significantly different than zero, which will indicate that there is a systematic change in subjects' choices across the two deprivation states.

For our subject-by-subject analysis we simply calculated, for each subject in each state and for every reward type, the proportion she chose the *lottery* option out of the total number of choices:
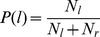
Where *P(l)* is the proportion a subject chose the *lottery* option for a given reward type in a given state. *N_l_* and *N_r_* are the number of times the subject chose the *lottery* and *reference* options, respectively. For examining significance effects across our subjects between the two states we used a repeated measure analysis of variance (ANOVA) with *P(l)* as the dependent variable and with State (*Satiated* and *Deprived*) and Reward-Type (*money*, *food* and *water*) as the repeated variables. P<0.05 was considered a significant effect.

### Parametric Method: Estimating Utility Functions

We used (random) expected utility theory in order to derive a utility function for each reward type in each session using the data from the same-type trials. We modeled the utility functions for each reward type as a power function having the form:
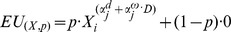
Where *p* is the stated probability that an option will yield a reward (*p* = 1.0 in *reference* options or the stated probability in *lottery* options), *X* is the objective value of the offered reward, 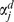 is the free parameter representing the level of risk aversion for specific reward type *j* in the deprived state, 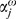 is the free parameter representing the addition (subtraction) to the level of risk for specific reward type *j* in the satiated state, and *D* is a dummy variable representing session type (deprived or satiated). Thus, we jointly estimated the risk levels for all three reward types using data from both sessions and looked for a systematic change in risk levels across sessions.

With this function, an *α* = 1 represents a risk neutral agent, an *α*<1 represents a risk-averse agent with a concave function and an *α*>1 represents a risk-seeking agent with a convex function. We selected this particular functional form for risk-aversion because of its simplicity, wide use in the literature, and because it accounted for a very high proportion of the variance in the choices measured in our study.

Using maximum likelihood estimation, the choice data for all reward types from both sessions of the same-type trials for the representative agent or for each subject were simultaneously fit to a single logistic function of the form:
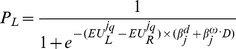
Where *P_L_* is the probability that the representative agent/subject chose the lottery option, 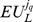 and 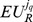 are the expected utility for the *lottery* and *reference* options for each reward type in each session *q*, respectively, 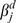 is the slope of the logistic function in the deprived state and 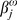 is the addition (subtraction) to the slope for specific reward type *j* in the satiated state, and *D* is a dummy variable representing session type (deprived or satiated).

Again, as in the non-parametric case, we tested to see if the coefficients of the state parameter *D* (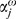 and 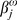) in the regression were significantly different than zero, which would indicate that there was a systematic change in subjects' risk preferences or the stochasticity of their choices, respectively, across the two states. For the subject-by-subject analysis we compared the fitted risk parameters (*α_j_*) between the two states using the Wilcoxon signed-rank test (a non-parametric test for comparing medians).

### Mixed-Type Trials: Estimating the “Behavioral Scaling Factor”

Using the fitted parameters from the same-type trials and the choice data from the mixed-type trials we estimated the relative pricing between money and food and water for each subject. (These parameters were fit after utility functions were fixed because our dataset did not provide sufficient power to support the simultaneous fitting of all parameters, a procedure we have used previously; [8]). We introduced here a linear factor that scaled the expected utility of food and water to that of money in a manner that predicted choice. We thus effectively searched for families of indifference points where:
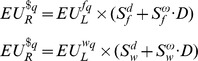
Where 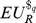 is the expected utility of the *reference* option in monetary subjective value units for each state *q*, 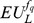 and 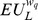 are the expected utilities of the *lottery* options in subjective value units of food and water, respectively and *q* represents the relevant state (deprived or satiated). 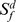 and 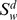 are the fitted free parameters scaling factors for food and water *lotteries*, respectively during the deprived state, 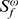 and 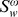 are the addition (subtraction) to the scaling factors for food and water in the satiated state, and *D* is a dummy variable representing session type (deprived or satiated). Again, the free parameters were fit using maximum likelihood estimation. The choice data for each reward type (food and water) from both sessions of the mixed-type trials for the representative agent or for each subject were simultaneously fit to a single logistic function of the form:
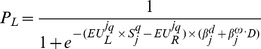
Where *P_L_* is the probability that the subject chose the lottery option.

For all the fitted models, we estimated how well our model fit the data with a pseudo-R^2^ value computed as the ratio between the log-likelihoods of the fits obtained to the observed choices and the log-likelihood of the fit that would have been obtained from a completely random chooser.

### Assessing the Degree of Variability in Risk Preferences Across the Population

In order to examine if there was a difference between the variance of stated and hungry risk attitudes across our population, the distributions of either the percentage of *lottery* option choices or the fitted risk parameters were examined. We bootstrapped the standard errors of these distributions in each state 1000 times and computed the mean and standard deviation of the resulting distributions. We then conducted a Wilcoxon signed-rank test (a non-parametric test for comparing medians) on those new distributions to examine if the mean standard deviation between the states was different (a P<0.05 was considered significant).

## Results

### Manipulation of State

In order to determine whether our deprivation protocol was successful, subjective hunger and thirst levels were assessed prior to each session using a visual analogue scale (VAS). Subjects reported significantly higher hunger and thirst levels during the deprived session than during the satiated session (paired t-test, P<0.0001 for both hunger and thirst, Fig. S1). Further, the number of subjects who always chose the consumable reward in mixed type trials, regardless of the quantity or probability of the consumable reward, increased starkly under conditions of deprivation. Fourteen percent of subjects exclusively chose the consumable reward when deprived (both for food and water), compared with 4% (food) and 5% (water) exclusively choosing the sure money option (Fig. S2). In contrast, almost none of the subjects in the satiated state (4% vs. 18% and 7% vs. 18% for food and water, respectively) chose the food and water rewards exclusively compared to choosing exclusively the sure money option (Fig. S2).

### Group Average: Representative Agent Analysis

#### Non-Parametric

We examined whether there was a systematic change in choice behavior as a function of internal state in the representative agent. We found that when combining all reward types, subjects chose the lottery option, on average, more often in the deprived state as compared to the satiated state (Table 1, Logit regression, p = 0.026). In addition, as can be seen in Table 1 there was a significant effect of State for choices over food (p = 0.028) and water (p = 0.036) and a non-significant trend for choices over money (p = 0.096). That is, the representative agent chose the lottery option significantly more times in the deprived state than in the satiated state when facing food and water choices and had a tendency to show the same effect for money options.

**Table 1:** Effect of state on risk behavior: Representative agent in the non-parametric approach.

Reward Type	Variable	Coef.	Robust Std. Err.	z	P>z	95% CI	
**All Rewards**	**State**	−0.17	0.07	−2.23	***0.026***	−0.33	−0.02
	**Constant**	−0.03	0.08	−0.40	0.686	−0.18	0.12
**Money**	**State**	−0.13	0.08	−1.66	0.096	−0.28	0.02
	**Constant**	0.22	0.08	2.80	0.005	0.07	0.38
**Food**	**State**	−0.18	0.08	−2.20	***0.028***	−0.34	−0.02
	**Constant**	−0.13	0.08	−1.61	0.108	−0.28	0.03
**Water**	**State**	−0.20	0.10	−2.09	***0.036***	−0.39	−0.01
	**Constant**	−0.18	0.09	−2.11	0.035	−0.36	−0.01

The results of the logit regression on choice for the representative agent for all reward types. Note that the *Constant* variable represents the deprived state and the *State* variable represents the addition (subtraction) during the satiated state. A **Bold** *Italic* font represents a significant effect of state. Coef. – the regression coefficient; Std Err – standard errors; z – z score of the regression; P>z – pvalue of the regression; CI – confidence interval.

#### Parametric: Utility Function Estimation

To assess the effect of a change in internal state on risk preferences we also estimated the utility functions for all reward types combined and for money, food and water separately using the pooled data from all subjects in both sessions. In order to examine the choice consistency in our data (essentially the transitivity of our subjects) we separated the data first by reward type and then by probability of the lotteries. This allowed us to examine the probability (out of the 6 repetitions of each choice option) that on average the representative agent would choose the risky option as a function of reward magnitude. We found that on average the likelihood to select the lottery option over the certain option varied as a lawful function of the magnitude of the risky reward for all probabilities (Fig. S3, S4). This demonstrates that subjects were, on average, consistent in their choices and were sensitive to both magnitude and probability of reward.

Note that, expected utility approaches, by design, can only be applied when subjects are technically consistent in their choice behavior. While it is unarguably true that subjects in the real world are often inconsistent, it is also unarguably true that when a chooser is being consistent [13], [14] the most compact description of the subject's risk attitude is with a utility function. We thus checked for consistency in these choices not as a theoretical statement but simply to confirm that the risk model we were using could be effectively applied under these conditions.

As shown in Table 2, the parameter α, the curvature of the utility function, takes a significantly lower value under satiation for all reward types. This is reflected by the fact that coefficient of the State parameter (*D*) was significantly different than zero, which indicates that, *on average*, our population was less risk-averse when deprived than when sated (the constant parameter is for the deprived condition). When people are moderately hungry and thirsty, overall, they are more risk tolerant in their decisions about food, water, and for monetary rewards. Recall that an α value <1 indicates risk aversion with an α = 1 indicating risk neutrality. Under conditions of satiation the average α value for all reward types combined was equal to 0.43 (and separately 0.59, 0.50 and 0.45 for money, food and water, respectively). Under deprivation this average value shifted up to 0.51. Similar to the non-parametric approach, even when separating the reward types all risk parameter values shifted up to 0.65, 0.58 and 0.54 for money, food and water, respectively. To our knowledge this is the first time that changes in risk preferences as a function of internal state have been shown to occur across multiple reward types simultaneously, and the first indication that risk attitudes to all three reward types change in a similar manner.

**Table 2:** Effect of state on risk behavior: Representative agent in EUT approach.

	Reward Type	Variable	Coef.	Robust Std. Err.	z	P>z	95% CI	
**Alpha**	**All Rewards**	**State**	−0.08	0.02	−3.17	***0.002***	−0.12	−0.03
		**Constant**	0.51	0.03	18.38	0.000	0.45	0.56
	**Money**	**State**	−0.06	0.03	−2.04	***0.041***	−0.12	0.00
		**Constant**	0.65	0.03	18.66	0.000	0.58	0.72
	**Food**	**State**	−0.08	0.03	−2.89	***0.004***	−0.14	−0.03
		**Constant**	0.58	0.03	19.98	0.000	0.52	0.64
	**Water**	**State**	−0.09	0.03	−2.84	***0.005***	−0.15	−0.03
		**Constant**	0.54	0.03	16.83	0.000	0.47	0.60
**Beta**	**All Rewards**	**State**	0.06	0.05	1.18	0.239	−0.04	0.16
		**Constant**	0.29	0.04	7.08	0.000	0.21	0.37
	**Money**	**State**	0.01	0.07	0.18	0.859	−0.13	0.15
		**Constant**	0.81	0.06	13.22	0.000	0.69	0.93
	**Food**	**State**	−0.03	0.08	−0.42	0.677	−0.18	0.12
		**Constant**	0.75	0.07	11.27	0.000	0.62	0.88
	**Water**	**State**	−0.23	0.12	−1.86	0.063	−0.48	0.01
		**Constant**	1.93	0.11	17.58	0.000	1.71	2.14

The values of the fitted risk parameters (using maximum likelihood estimation) as a function of state for the representative agent for all reward types. Note that the *Constant* variable represents the value in the deprived state and the *State* variable represents the addition (subtraction) during the satiated state. A **Bold** *Italic* font represents a significant effect of state. Alpha – the fitted risk parameter. Beta – the slope of the logit function. Coef. – the regression coefficient; Std Err – standard errors; z – z score of the regression; P>z – pvalue of the regression; CI – confidence interval.

Next we examined the effect deprivation has on the values subjects placed on food and water relative to money. To accomplish this we pooled mixed-type trials and estimated the representative agent's food and water scaling factors as detailed in the Methods section. We thus determined, for each state, what amount of food (and water) was equal in value to a sure gain of $0.50 across the range of food and water reward probabilities and magnitudes that we examined above. As can be seen in Table 3, deprivation increased the value of food relative to money as measured by the scaling factors (from 0.20 to 0.26, P = 0.06). However, the value of water relative to money did not change significantly as a function of a change in internal state (P>0.05).

**Table 3:** Effect of state on relative value: Representative agent in EUT approach.

	Reward Type	Variable	Coef.	Robust Std. Err.	z	P>z	95% CI	
**Scale**	**Food**	**State**	−0.07	0.03	−2.09	***0.037***	−0.13	0.00
		**Constant**	0.26	0.04	7.49	0.000	0.19	0.33
	**Water**	**State**	0.01	0.01	0.91	0.365	−0.01	0.02
		**Constant**	0.06	0.01	7.47	0.000	0.04	0.07
**Beta**	**Food**	**State**	0.88	0.26	3.33	***0.001***	0.36	1.39
		**Constant**	1.36	0.23	5.90	0.000	0.91	1.81
	**Water**	**State**	0.80	0.24	3.27	***0.001***	0.32	1.28
		**Constant**	1.38	0.21	6.48	0.000	0.96	1.79

The values of the fitted scaling factors (using maximum likelihood estimation) for food and water relative to money as a function of state for the representative agent. Note that the *Constant* variable represents the value in the deprived state and the *State* variable represents the addition (subtraction) during the satiated state. A **Bold** *Italic* font represents a significant effect of state. Scale – the fitted scaling factor. Beta – the slope of the logit function. Coef. – the regression coefficient; Std Err – standard errors; z – z score of the regression; P>z – pvalue of the regression; CI – confidence interval.

It should be noted, however, that the scaling factor is effectively a measure of relative value in utility space not in value space (see Methods). Direct comparison of the utilities of a given monetary and food reward pair requires the combination of information from the risk parameters (*α_s_*) and the scaling factors and comes with several assumptions. It is possible that a real change in the relative monetary value of water did manifest itself uniquely in the scaling factor because of the deprivation-related change in α (Figure S5).

### Within-Subject Analysis

We next conducted a within subject analysis of the average change in risk preferences as a function of state.

#### Non-Parametric

**Figure 2:**
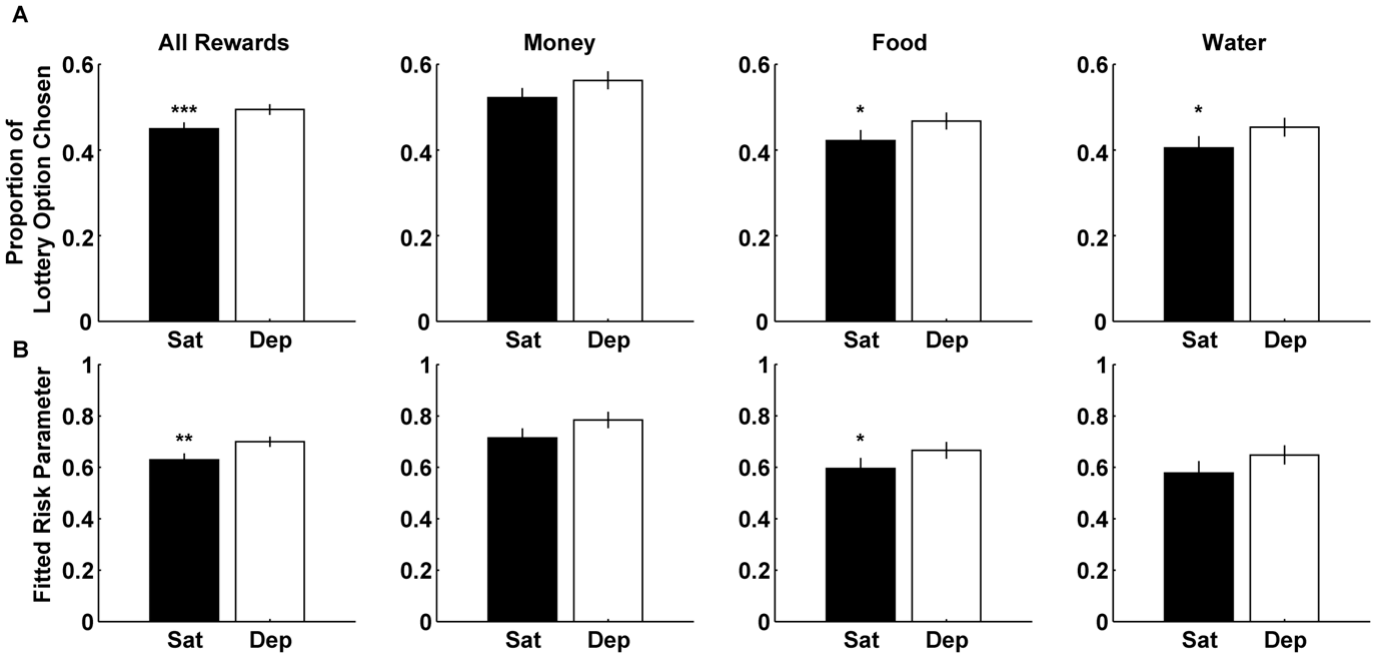
Average effect of state on risk behavior: Within-subject. A) The average (across subjects) of the proportion to choose the lottery option out of the total number of choices made in both states for all reward types. B) The average (across subjects) of the fitted risk parameters (*α*) in both states for all reward types. Data represents the mean ± s.e.m. * p<0.05; ** p<0.002; *** p<0.0001.

As can be seen in figure 2A, when combining all choices across all reward types, subjects chose the lottery option 49%±1.8% (s.e.m) out of the total number of choices in the deprived state compared to only 45%±2.3% (s.e.m) in the satiated state (Repeated measure ANOVA, F_1,162_ = 13.1, p<0.0001). A similar pattern was evident when we analyzed separately the choices for each of the reward types. The proportion subjects who chose the lottery option in the deprived state was significantly higher than in the satiated state for food (F_1,54_ = 5.1, p = 0.028) and water (F_1,54_ = 4.56, p = 0.037) and there was a trend towards a significant effect for money (F_1,54_ = 3.56, p = 0.064). This indicates that subjects demonstrated a higher propensity to choose the lottery option in the deprived state than in the satiated state. Note, however that the effect size is rather small. It is only in the order of a 5% change. We address this issue further below.

#### Parametric: Utility Function Estimation

We estimated the utility functions for money, food and water separately for each subject in both states. In a similar manner to the non-parametric approach, we compared the average fitted risk parameters of our subjects in the two states. Using EUT gave very similar results to the non-parametric method. As can be seen in Figure 2B, combining the fitted risk parameters of all reward types revealed that on average subjects were significantly less risk averse in the deprived state compared to the satiated state (Wilcoxon rank test; n = 162, z = −3.2, p = 0.001). The average fitted risk parameter across all reward types and subjects in the satiated condition was α = 0.63±0.025 (s.e.m) and it increased (meaning less risk aversion) in the deprived condition to α = 0.70±0.02. Furthermore, when separating the reward types, the average fitted risk parameter in the deprived state was higher than in the satiated state for food (Wilcoxon rank test; n = 54, z = −1.96, p = 0.05) and there was a marginally significant effect for money (Wilcoxon rank test; n = 54, z = −1.82, p = 0.069) and a trend in the same direction for water (Wilcoxon rank test; n = 54, z = −1.68, p = 0.092). Note again, however that the effect size of deprivation is rather small. We address this issue further below.

#### Convergence of Risk Attitudes Under Deprivation

The fact that we observed only a small effect of internal state on risk preference (regardless of how we measured it) raises the possibility that there is a more complicated interaction between internal state and risk preferences. To explore this possibility, we examined the distribution of risk preferences in the two states across our population to see if we could find any systematic change using a linear regression that correlated the proportion that each subject chose the lottery option (out of the total number of choices) in the satiated state to that in the deprived state. For completeness, we conducted the same regression while correlating subject-specific risk preferences (fitted α) in the satiated state to the risk preferences in the deprived state across the individuals in our sample.

**Figure 3:**
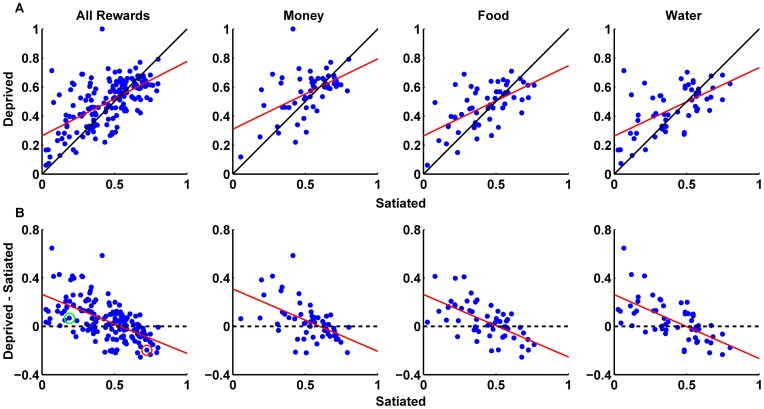
Correlation of risk behavior between states: Non-parametric. (A) Correlations between the proportions to choose the lottery option in the satiated state and in the deprived state across all subjects for all reward types. Each point represents the proportions in both states for a single subject. (B) Correlations between the proportions to choose the lottery option in the satiated state and the difference in proportions (deprived - satiated) across all subjects for all reward types. The black line represents the unity line. The red line represents the least square fit. The green circle highlights an example subject that increased her proportion to choose the lottery option when in the deprived state. The red circle highlights an example subject that decreased her proportion to choose the lottery option when in the deprived state. Details regarding the regression in A can be found in Table 1.

**Figure 4:**
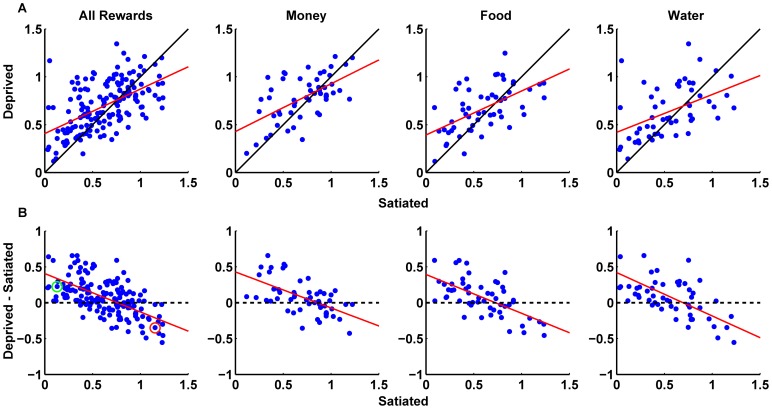
Correlation of risk behavior between the to states: EUT. (A) Correlations between the fitted risk parameters in the satiated state and in the deprived state across all subjects for all reward types. Each point represents the fitted risk parameters in both states for a single subject. (B) Correlations between the fitted risk parameters in the satiated state and the difference in the risk parameters (deprived - satiated) across all subjects for all reward types. The risk parameters for all reward types in each subject were jointly estimated. The black line represents the unity line. The red line represents the least square fit. The green circle highlights an example subject that became less risk averse when in the deprived state. The red circle highlights an example subject that became more risk averse when in the deprived state. Details regarding the regression in A can be found in Table 2.

As shown in Table 4, the regression coefficients for all three reward types (for both analytic approaches) were positive and significant, indicating that there was a systematic change in risk attitude for all reward types between the two states. However, the direction of the change in risk preferences is not at all straightforward. As can be seen in Figures 3A and 4A some of the values are located above the unity line indicating that these values increased in the deprived condition relative to the satiated condition. On the other hand, some of the values are located below the unity line indicating that these values decreased in the deprived condition relative to the satiated condition. Note that the regression line intercepts the unity line at values ranging from 0.5–0.6 for the non-parametric analysis and 0.69–0.85 for the EUT analysis depending on the reward type (see Table 4).

**Table 4:** Regression values (see Figures 3 and 4).

Non-Param	B_0_	B_1_	T value	P value	Intercept Point (x = y)
**All Rewards**	0.26 (0.03)	0.51 (0.05)	9.74	<0.000001	0.54
**Money**	0.31 (0.06)	0.48 (0.11)	4.37	5.90E-05	0.60
**Food**	0.26 (0.04)	0.48 (0.09)	5.46	1.29E-06	0.51
**Water**	0.26 (0.04)	0.47 (0.09)	5.42	1.48E-06	0.50

EUT	B_0_	B_1_	T value	P value	Intercept Point (x = y)
**All Rewards**	0.41 (0.04)	0.47 (0.05)	8.66	5.00E-15	0.76
**Money**	0.43 (0.07)	0.50 (0.10)	5.19	3.52E-06	0.85
**Food**	0.39 (0.06)	0.46 (0.09)	5.00	6.91E-06	0.73
**Water**	0.42 (0.06)	0.40 (0.10)	4.12	1.36E-04	0.69

The values of the regressions conducted on the proportion to choose the lottery option (Non-Param) and on the fitted risk parameters (EUT) between the two states across all subjects for all reward types. B0 – intercept; B1 – regression coefficient; T Value – the t-statistic of B1; P Value – the p value of the t-statistic; Intercept Point – the intercept point of the regression line with the unity line, i.e. when the values in the two states are equal.

To determine if this deviation from the unity line is due to a systematic effect, we computed for each subject, for all reward types, the difference between the proportion a subject chose the lottery option in the deprived state and the proportion a subject chose the lottery option in the satiated state. We then plotted this difference against the proportion the subject choose the lottery option in the satiated state (Figure 3B). For completeness we conducted the same analysis for the fitted risk parameters (Figure 4B).

As can be seen in Figures 3B and 4B, there is a significant but negative correlation across subjects in all reward types. Note, that we did not alter the data values in any way, and that the point where the regression line crosses the x-axis is identical to the crossing point with the unity line in the original regression as indicated in the regression parameters described in Table 3. The points that have a value of zero on the y-axis represent subjects with risk preferences that did not change between the two states. Points that have a positive value on the y-axis (like the dot surrounded by a green circle) represent subjects that in the satiated state did not choose the lottery option very often (had a low proportion of choosing the lottery option and had a low alpha and therefore were very risk averse) but increased their propensity to choose the lottery option (increased their proportion of choosing the lottery option and had a higher alpha value and therefore were less risk averse) when moving to the deprived state. On the other hand, points that have a negative value on the y-axis (like the dot surrounded by a red circle) represent subjects that in the satiated state chose the lottery option very often (had a high proportion of choosing the lottery option and had a high alpha and therefore were risk tolerant or seeking) but decreased their propensity to choose the lottery option (decreased their proportion of choosing the lottery option and decreased their alpha value and therefore became more risk averse) when moving to the deprived state. The analysis shows us that, on average, the point at which the regression line crosses the x-axis is the point that splits the data in terms of the effect satiation has on subjects' behavior.

Stating it in another way, subjects that did not like risk in the satiated state tend to become less risk averse in the deprived state. In contrast, subjects that liked to take gambles in the satiated state tended to become less so in the deprived state. Hence, it appears that under deprivation subjects converge towards a similar level of risk preference, which according to our analysis is one of moderate risk aversion.

Note that this convergence effect was not mediated by an effect of internal state on the stochasticity of subject choices; it is not simply that hungry subjects are more random. As can be seen in Table 2, the noise parameter in our model fits (*β*) did not systematically change as a function of state. In addition, we have looked into the possibility that the starting time of the experiment may have had an effect on our results but we did not find any significant effects (data not shown).

**Figure 5:**
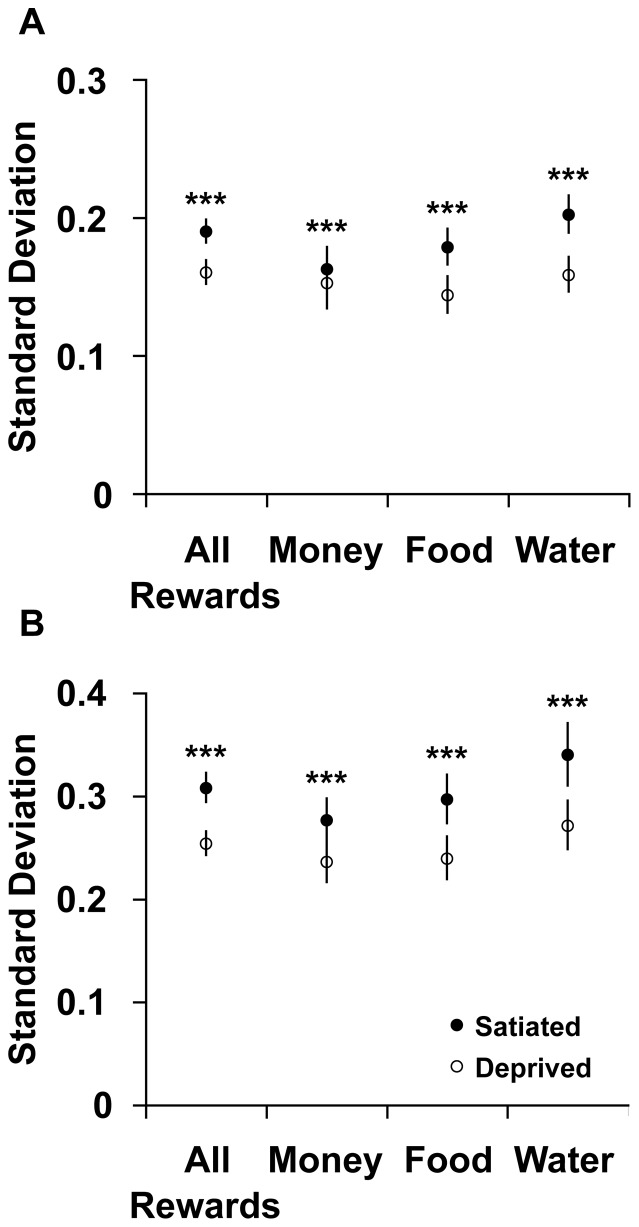
Degree of variance. A) Standard deviation (STD) in the non-parametric approach. B) Standard deviation in the EUT approach. The average standard errors of the distributions across subjects of the proportion to choose the lottery option (A) and the fitted risk parameters (B) for all reward types in both states. A lower average standard error demonstrates convergence towards a similar value and lower variance in the deprived condition. For comparing the averages and conducting a significance test, we bootstrapped the standard errors across subjects for all reward types in each state. Note that the data represents mean ± STD (and not s.e.m.). *** p<0.0001.

In order to further test the conclusion risk preferences across subjects converge towards a single value under deprivation, and to rule out the possibility that our result is due to convergence (or regression) to the mean, we assessed the degree of variance in risk attitudes across our subjects for each reward in each state. If subjects really do converge towards a common risk attitude under deprivation, then the variance across subjects in the deprived state should be smaller than in the satiated state. This would not be the case if the phenomenon that we observed was an example of regression towards the mean. As indicated in Figure 5, this was observed for all reward types for both the non-parametric and parametric analyses (p<0.001, bootstrap standard errors).

It thus appears that while the *average subject* does increase the propensity with which they choose the lottery option under deprivation as has been previously observed, this is only because more subjects tend to dislike risk when sated. According to our data, individual subjects do not all become more risk loving when deprived as has been previously suspected, but rather they appear to converge toward a similar degree of moderate risk aversion for all reward types when deprived.

## Discussion

Our results confirm and extend previous findings but in a novel, and perhaps theoretically important, way. Symmonds and colleagues [11] found that, overall, meals which had a small effect on satiety (as assessed by plasma Ghrelin levels) led on average to increases in monetary risk tolerance while largely sating meals led, on average, to increases in monetary risk aversion. We found that, across our entire population, humans become overall more risk tolerant (less risk averse) as they become overall hungrier and thirstier, a finding reasonably well aligned with this and other previous studies [15]–[17]. Interestingly, we found that this was true not just with regard to monetary decisions but also with regard to decisions about food and water.

More interesting, however, and contrary to our initial predictions, were our within-subjects observations. We found that when sated, individual human subjects showed very diverse risk attitudes, ranging from being highly risk-averse to being weakly risk tolerant. When deprived these risk attitudes converged towards a similar level, *for all reward types*, which could be described as weakly risk averse. Our results suggest that risk preferences are indeed state-dependent but in a more complicated way than had been previously suspected.

Although consistent with previous findings, our results from the representative agent analysis showing an increase in risk tolerance under deprivation should be interpreted with caution, given our within-subject findings. Our within-subject data suggest, in essence, that any characterization of the representative agent necessarily reflects the structure of the sample population – as would any such measurement – and is very sensitive to that structure. Previous studies have shown that most subjects are risk averse in tasks of these kinds [18]–[20]. Therefore, when conducting a simple average across all subjects, the majority (people who are risk averse while satiated) will dominate this measurement. These people tend to become more risk tolerant when deprived. This masks the effect of the minority, people who are risk seeking while satiated, and tend to become more risk averse when deprived. Our data suggest that the key feature of deprivation is that it reduces the variance of human risk attitudes in a population.

However, we do wish to emphasize that our experimental method and payment mechanism is not the same as it is usually implemented in animal experiments studying risk. Our subjects made hundred of choices but were rewarded on the basis of only one randomly chosen trial (for each reward type) at the end of the experiment. Although this approach is incentive compatible and standard practice in behavioral economics (e.g. [21]) it is not the same as the animal experiments in which each trial might be rewarded (depending on the probabilities). Therefore, in order to generalize our findings to other species, further studies need to be conducted in humans/animals using similar methods as in the animal literature.

We emphasize that our convergence effect in deprivation holds not only for decisions about money but also for decisions about food and water. In a previous study, we showed that in a mildly deprived state within-subject risk attitudes are highly correlated suggesting the existence of a common valuation system [12]. This high correlation was still evident in the current study, in both states, strengthening the notion of a common valuation system that is activated in both internal states (see also [22]).

At an evolutionary level, one might hypothesize that in the past, groups of humans have been exposed to a range of environments ranging from those in which resource were plentiful to those in which resources were scarce. Under scarce resource conditions that moderately increase mortality rates, risk attitudes might be expected to impact more directly on survival than under conditions of plenty. One possibility that might be worth considering is that under these conditions of moderate scarcity, animals may be driven towards more homogeneous risk preferences, a phenomenon that was evident in our data. But we stress that this is, of course, only a speculation.

Previous studies have demonstrated an interaction between money and food and internal state on subject preferences. Men who feel either poor or hungry, for example, prefer heavier women than do men who feel rich or satiated [23]. In a similar vein, hungry subjects donate less to charity and to other players in a “give-some game” than do their sated peers, and they eat more chocolate M&M's after imagining winning €25 than after imagining winning €25,000 [24]. There is significant evidence of an interaction between satiation levels and financial preferences. Feeling hungry may be similar to feeling poor and vice versa. This hints at the existence of a common valuation network in the brain, a single mechanism for valuing many different kinds of rewards that originated long before fiat currencies were introduced in modern civilizations.

Is there evidence that, as whole populations shift from sated to deprived state, the structure of human decision-making changes? Some evidence from stock market behavior in Muslim countries may, in light of our findings, suggest that this is the case. During the Muslim holy month of Ramadan, observant Muslims refrain from food and water during daylight hours. If our observations at the individual level are correct, then one might plausibly expect that stock market behavior in Muslim countries could be significantly impacted by the food deprivation that occurs during Ramadan. In fact, existing papers [25], [26] suggest that this is the case. Studies of Muslim stock markets indicate that overall market volatility declines sharply during the month of Ramadan, a macroeconomic effect that is compatible with our microeconomic observations. Of course these observations also suggest that decision-making by populations under deprivation due to war, famine and geopolitical conflict may be systematically altered in predictable ways that may be of importance to policy makers.

The use of fiat currencies, which hold no intrinsic value, and the fact that we constantly make choices under conditions of mixed rewards, i.e. money vs. primary rewards, in different internal states, raises unique questions about risk behavior and the representation of value in the brain. It is not only because the brain must evaluate these rewards against a common currency [12], [22], [27]–[31] but also because people reach satiation points with food and water but not with money, and unlike other organisms, humans ubiquitously employ currency as a secondary reward for resources.

## Supporting Information

Figure S1 **Hunger and thirst ratings.** Subjective hunger and thirst levels were assessed prior to each session using a visual analogue scale (VAS). A within-subjects analysis of the VAS ratings across sessions revealed a significant effect of state in both hunger and thirst (paired t-test, P<0.0001). This indicates that subjects reported higher levels of hunger and thirst in the deprived state as compared to the satiated state.
(TIF)

Figure S2 **Corner solvers during mixed-type trials.** The proportion of subjects that did not show any behavioral variation during the mixed-type trials is displayed. These subjects only chose one reward type throughout the session for money/food (left) and money/water (right) options in both states. Reference – subjects who chose the sure amount of $0.5 in all trials. Lottery – subjects who chose the lottery option (across all reward magnitudes and probabilities) in all trials. There is a strong effect of state on the proportion of corner solvers. A higher proportion of subjects only chose the reference option during the satiated state while the opposite was true for the deprived state; a higher proportion of subjects only chose the lottery options.
(TIF)

Figure S3 **Representative agent's choice data and fit in same-type trials: Satiated state.** Top: Choice data for the representative agent from the same-type trials for money (left), food (M&M's and Ritz, middle) and water (right). Each dot represents the probability the agent chose the *lottery* option as a function of the reward magnitude of the lottery option. The colors represent the five different winning probabilities of the lottery option. All the dots for a given winning probability (same color) are connected with a dotted line for clarity. The solid lines represent the best-fitted logit using maximum likelihood estimation with risk aversion (*α*) and the slope (*β*) of the logit function as free parameters. n, represents number of trials. Bottom: Utility functions derived from the choice data and fit for the representative agent for all reward types. The utility functions simply plot the psychophysical curves that relate objective reward magnitude to the perceived subjective value required to account for the observed choice behavior. The blue line represents the mapping between the objective values (X axis) to the subjective values (Y axis) using the fitted risk aversion parameter (*α*) for each reward type and a utility function in the form of Y = X*^a^*. The different values of *α* represent the average values of fitted risk aversion for all reward types.
(TIF)

Figure S4 **Representative agent's choice data and fit in same-type trials: Deprived state.** Same as figure S3 but in the deprived state.
(TIF)

Figure S5
**Risk parameters and scaling factors.** The values of the scaling factors (represented as a color map) are represented as a function of the interaction between the values of risk parameters for money and food and different reward magnitudes. X_money_ – amount of money. X_food_ – amount of food. The formula for calculating the scaling factor is: 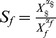.(TIF)
