# Supplementary figures and images for: State Dependent Valuation: The Effect of Deprivation on Risk Preferences
Source: PLoS One. 2013 Jan 24;8(1):e53978. doi: 10.1371/journal.pone.0053978 (PMC3554724; doi:10.1371/journal.pone.0053978)

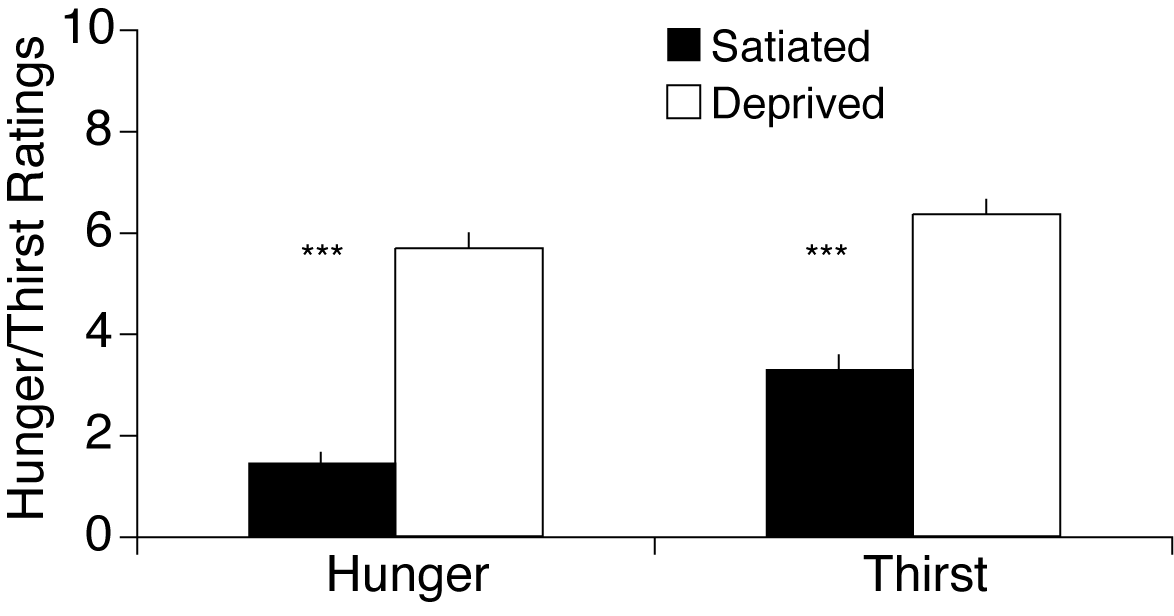

Supplement: Figure S1 — Hunger and thirst ratings. Subjective hunger and thirst levels were assessed prior to each session using a visual analogue scale (VAS). A within-subjects analysis of the VAS ratings across sessions revealed a significant effect of state in both hunger and thirst (paired t-test, P<0.0001). This indicates that subjects reported higher levels of hunger and thirst in the deprived state as compared to the satiated state. (TIF) [file pone.0053978.s001.tif]

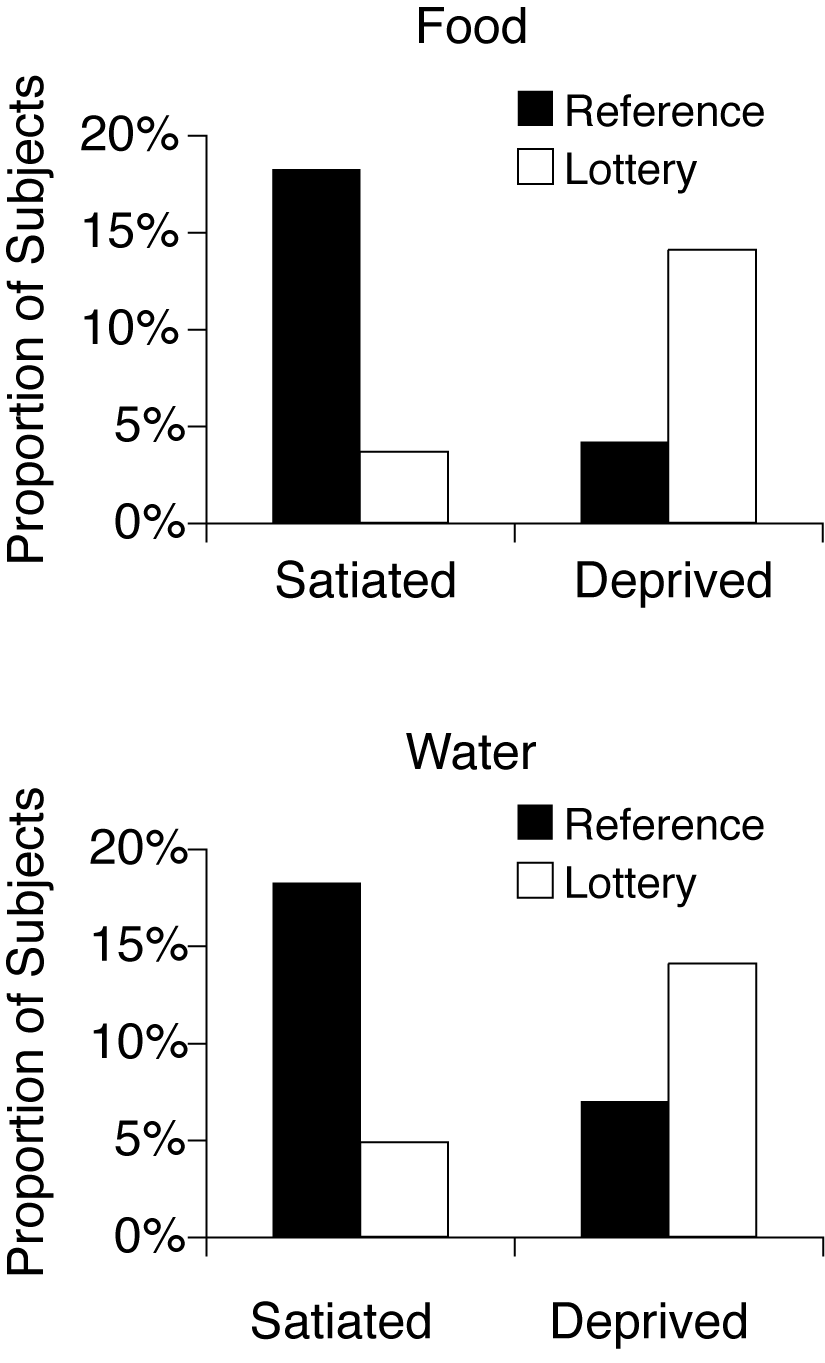

Supplement: Figure S2 — Corner solvers during mixed-type trials. The proportion of subjects that did not show any behavioral variation during the mixed-type trials is displayed. These subjects only chose one reward type throughout the session for money/food (left) and money/water (right) options in both states. Reference – subjects who chose the sure amount of $0.5 in all trials. Lottery – subjects who chose the lottery option (across all reward magnitudes and probabilities) in all trials. There is a strong effect of state on the proportion of corner solvers. A higher proportion of subjects only chose the reference option during the satiated state while the opposite was true for the deprived state; a higher proportion of subjects only chose the lottery options. (TIF) [file pone.0053978.s002.tif]

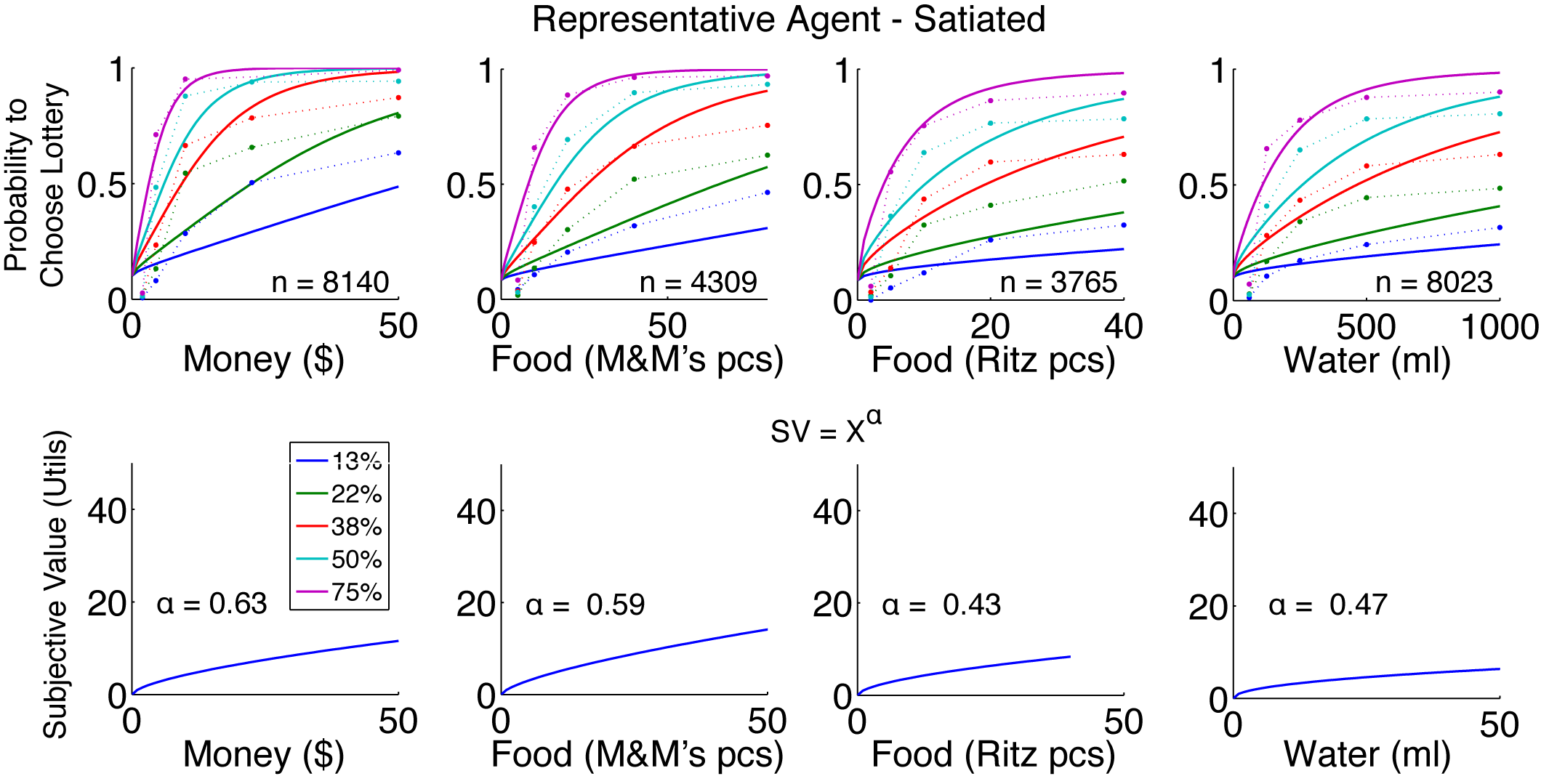

Supplement: Figure S3 — Representative agent's choice data and fit in same-type trials: Satiated state. Top: Choice data for the representative agent from the same-type trials for money (left), food (M&M's and Ritz, middle) and water (right). Each dot represents the probability the agent chose the lottery option as a function of the reward magnitude of the lottery option. The colors represent the five different winning probabilities of the lottery option. All the dots for a given winning probability (same color) are connected with a dotted line for clarity. The solid lines represent the best-fitted logit using maximum likelihood estimation with risk aversion (α) and the slope (β) of the logit function as free parameters. n, represents number of trials. Bottom: Utility functions derived from the choice data and fit for the representative agent for all reward types. The utility functions simply plot the psychophysical curves that relate objective reward magnitude to the perceived subjective value required to account for the observed choice behavior. The blue line represents the mapping between the objective values (X axis) to the subjective values (Y axis) using the fitted risk aversion parameter (α) for each reward type and a utility function in the form of Y = Xa. The different values of α represent the average values of fitted risk aversion for all reward types. (TIF) [file pone.0053978.s003.tif]

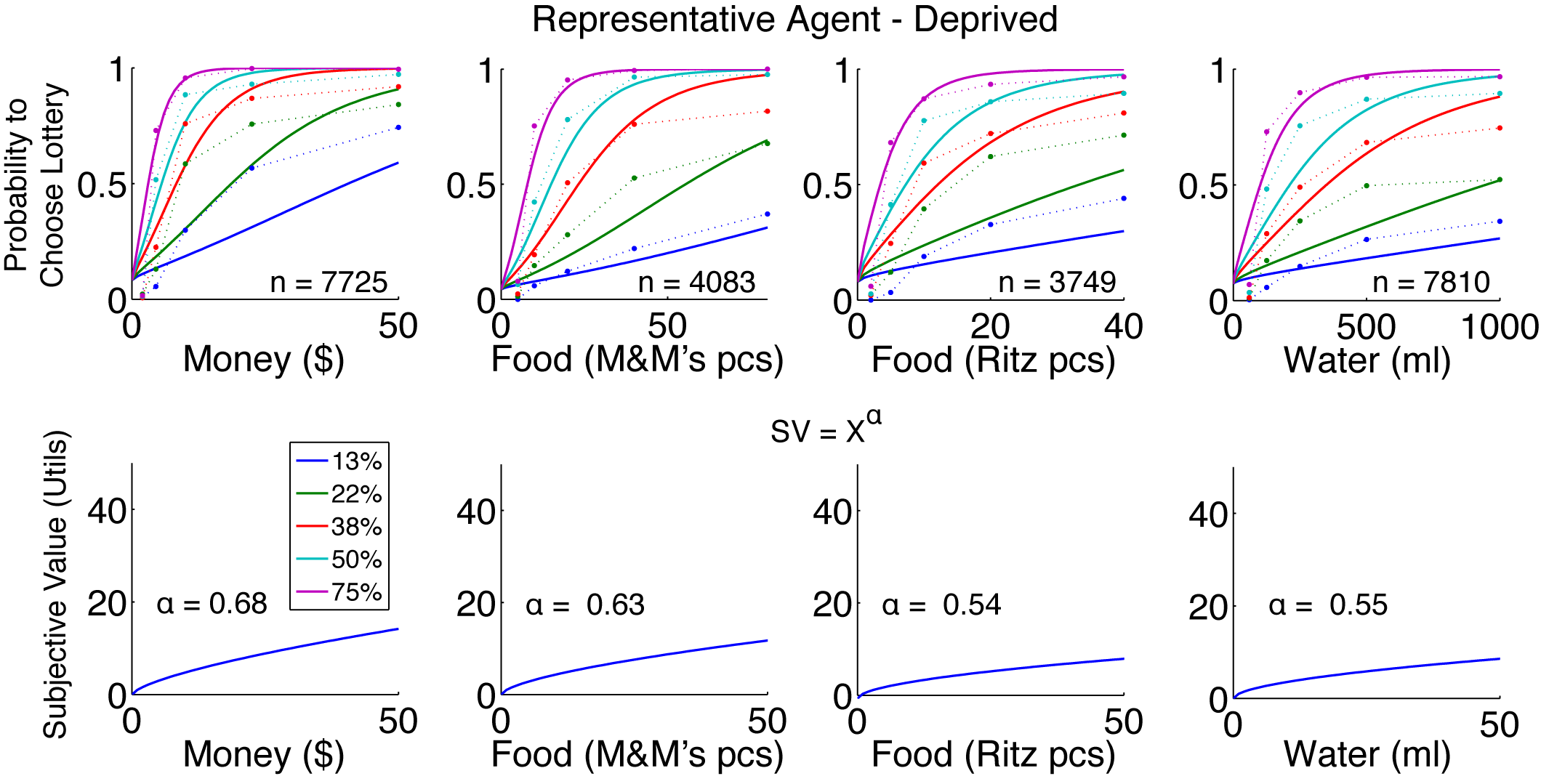

Supplement: Figure S4 — Representative agent's choice data and fit in same-type trials: Deprived state. Same as figure S3 but in the deprived state. (TIF) [file pone.0053978.s004.tif]

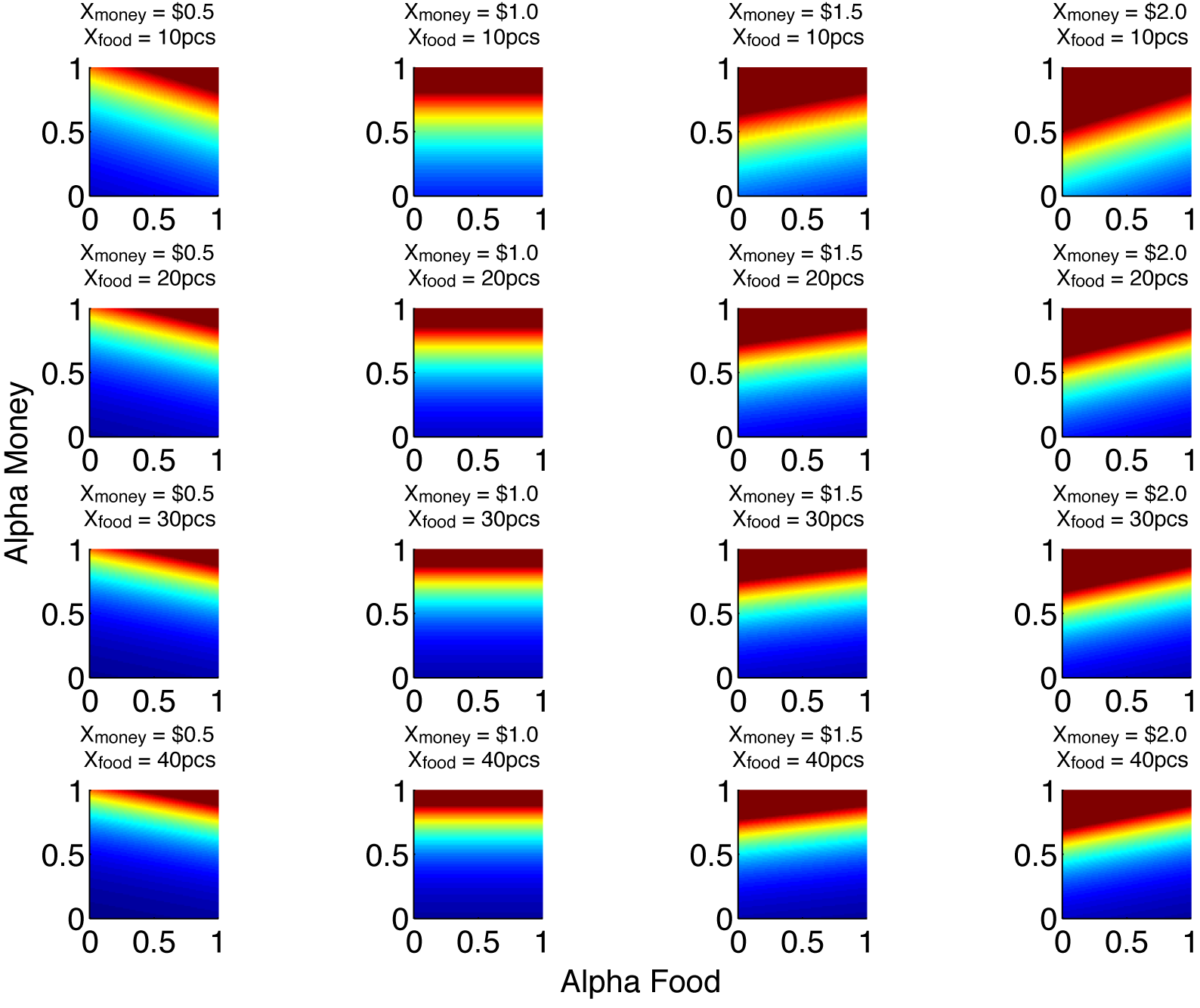

Supplement: Figure S5 — Risk parameters and scaling factors. The values of the scaling factors (represented as a color map) are represented as a function of the interaction between the values of risk parameters for money and food and different reward magnitudes. Xmoney – amount of money. Xfood – amount of food. The formula for calculating the scaling factor is: . (TIF) [file pone.0053978.s005.tif]
